# EGCG Attenuates Renal Damage via Reversing Klotho Hypermethylation in Diabetic db/db Mice and HK-2 Cells

**DOI:** 10.1155/2020/6092715

**Published:** 2020-08-27

**Authors:** Xiu Hong Yang, Bao Long Zhang, Xiao Meng Zhang, Jin Dong Tong, Yan Hong Gu, Li Li Guo, Hui Min Jin

**Affiliations:** ^1^Division of Nephrology, Shanghai Pudong Hospital, Fudan University, Pudong Medical Center, 2800 Gong Wei Road, Shanghai, China; ^2^The Institutes of Biomedical Sciences (IBS), Fudan University, 130 Dongan Road, Shanghai, China; ^3^Division of Vascular Surgery, Shanghai Pudong Hospital, Fudan University, Pudong Medical Center, 2800 Gong Wei Road, Shanghai, China; ^4^Hemodialysis Center, Bao Shan Branch of No. 1 People's Hospital, Shanghai Jiao Tong University, Shanghai, China

## Abstract

To explore whether epigallocatechin-3-gallate (EGCG) improves renal damage in diabetic db/db mice and high-glucose- (HG-) induced injury in HK-2 cells by regulating the level of Klotho gene promoter methylation. Western blotting was used to detect the protein expression levels of DNA methyltransferase 1 (DNMT1), DNMT3a, DNMT3b, transforming growth factor-*β*1 (TGF-*β*1), *α*-smooth muscle actin (*α*-SMA), and Klotho. The methylation level of the Klotho gene promoter was detected by pyrosequencing. Chromatin immunoprecipitation was used to detect the binding of the Klotho gene promoter to DNMT1 and DNMT3a. The expression of oxidative stress markers (reactive oxygen species (ROS), superoxide dismutase (SOD), malondialdehyde (MDA), catalase (CAT), and 8-hydroxy-2′-deoxyguanosine (8-OHdG)) and inflammatory cytokines (interleukin-1*β* (IL-1*β*), IL-6, and tumor necrosis factor-*α* (TNF-*α*)) in kidney homogenates was also measured using ELISA. Klotho and DNMT3b protein expression was upregulated, while DNMT1, DNMT3a, TGF-*β*1, and *α*-SMA protein expression was downregulated after EGCG treatment. EGCG treatment also reduced the methylation level of the Klotho gene promoter as well as the binding of DNMT3a to the Klotho gene promoter. In addition, EGCG treatment significantly decreased the levels of ROS, MDA, 8-OHdG, IL-1*β*, IL-6, and TNF-*α* and increased the levels of CAT and SOD. Under HG conditions, EGCG regulated Klotho gene promoter methylation via DNMT3a and decreased the methylation level of the Klotho gene promoter, thereby upregulating the expression of the Klotho protein to exert its protective effect.

## 1. Introduction

Diabetic kidney disease (DKD) is a chronic microvascular complication of diabetes mellitus (DM); it is also a major cause of deaths in patients with chronic kidney disease (CKD) [1]. DNA methylation is an important epigenetic modification and plays an important role in the development and progression of DKD. In addition, DNA hypermethylation is one of the important factors leading to gene silencing, eventually decreasing the expression of corresponding proteins [2, 3]. It has been reported that high glucose (HG) causes abnormal DNA methylation [4]. In diabetes, abnormal DNA methylation is not only caused by hyperglycemia but also by reactive oxidative stress (ROS), inflammation, cytokines, and growth factors [4, 5]. Studies have demonstrated that the level of DNA methylation in patients with DKD is different from that in diabetic patients without kidney disease [6].

Klotho was accidentally discovered in 1997 by Kuro in the study of spontaneous hypertension and originally identified as an antiaging gene. Klotho, as an antioxidative, antifibrotic, and anti-inflammatory molecule, is highly expressed in distal tubular epithelial cells and is closely associated with kidney diseases. In addition, the Klotho promoter is rich in CpG islands and vulnerable to DNA methylation [7–9]. Klotho DNA hypermethylation plays critical roles in the pathogenesis of kidney diseases. Recently, several studies have suggested that Klotho is closely associated with DKD. Lin et al. reported that the protein expression of Klotho was downregulated in the kidney tissues of streptozotocin- (STZ-) induced diabetic mice [10]. Moreover, it has been confirmed that decreased Klotho expression is closely related to increased proteinuria in db/db mice [11].

Epigallocatechin-3-gallate (EGCG), a kind of green tea extract, is the major polyphenolic constituent present in green tea [12]. At present, the studies on EGCG have been mainly focused on tumors and various metabolic diseases. EGCG could play an antitumor effect by inhibiting tumor cell proliferation and angiogenesis and promoting tumor cell apoptosis in the field of tumorigenesis [13–15]. To date, various studies have demonstrated that EGCG exhibits anti-inflammatory, antioxidative, and antifibrotic effects to play a protective role in metabolic diseases including diabetes and related complications, especially DKD [16–18]. However, the results of recent studies did not confirm the effects of EGCG on multiple signaling systems, and its target remains to be identified. Considering the similar function of EGCG and Klotho in kidney protection, we speculate that there is an association between EGCG and Klotho. Therefore, this study is aimed at investigating the renoprotective effects of EGCG and exploring whether EGCG exerts its protective effects via regulating Klotho expression in both db/db mice and HK-2 cells. Furthermore, we confirmed the relationship between EGCG and Klotho by transfecting HK-2 cells with Klotho overexpression (OE) and knockout (KO) vectors. Results were analyzed using pyrosequencing and chromatin immunoprecipitation (ChIP). Hence, an understanding of the epigenetic mechanisms underlying the regulation of Klotho expression by EGCG may help identify new targets for the treatment of DKD.

## 2. Materials and Methods

### 2.1. Animals

All animal procedures were approved by the Animal Care Committee of Fudan University. Six-week-old C57BLKS/J db/db mice and normal mice were obtained from the Model Animal Research Center of Nanjing University and housed in a specific pathogen-free animal facility at Pudong Hospital. All the mice had free access to normal rodent diet and distilled water. Prior to treatment, the 6-week-old mice were single-housed for adaptation for two weeks. Then, mice were allocated to the following groups (6 mice per group): normal group (nontreated C57 mice); control group (nontreated db/db mice); and EGCG group (db/db mice + EGCG). The mice of the normal and control groups were intragastrically administered with 0.9% saline, and the mice of EGCG group were intragastrically administered with EGCG 50 mg/kg/d (obtained from preliminary experiments). EGCG (No. E4143) was obtained from Sigma-Aldrich (St. Louis, MO, USA). The treatment lasted for 8 weeks. The graphical illustration of the mouse treatment plan is shown in Figure S8.

### 2.2. Cell Culture and EGCG Treatment

The human renal proximal tubular epithelial (HK-2) cell line and human embryonic kidney 293 cells (293) were purchased from the Bioresource Collection and Research Center and American Type Culture Collection, respectively. They were cultured in Dulbecco's Modified Eagle's Medium (Invitrogen, Carlsbad, CA, USA) supplemented with 10% fetal bovine serum (HyClone, South Logan, UT, USA). When HK-2 cells reached 80–90% confluency (at 37°C in 5% CO_2_/95% air atmosphere), they were maintained in a serum-free medium for 24 h. Next, HK-2 cells were incubated in medium supplemented with normal (5.5 mM) or high (60.0 mM) levels of glucose, and subsequently, cells were stimulated with EGCG (>95% purity; Sigma-Aldrich) at doses of 0.1, 1, 20, and 50 *μ*M in high-glucose condition for 24, 48, and 72 h.

### 2.3. Cell Viability

Cell viability was assessed using the Cell Counting Kit-8 (CCK-8) assay according to the manufacturer's instructions. The absorbance (OD value) was determined at 450 nm using an automated microplate reader (Infinite M200 PRO; Tecan, Männedorf, Switzerland).

### 2.4. Plasmid Construction and Klotho OE in HK-2 Cells

Total RNA was isolated from HK-2 cells using the TRIzol Reagent (Invitrogen, Carlsbad, CA, USA) and reverse transcribed into cDNA using the PrimeScript RT Reagent Kit with gDNA Eraser (Takara, RR047A). Next, we designed PCR primers and cloned Klotho gene fragments using Phusion High-Fidelity DNA Polymerase (M0530, NEB, MA, USA) (as shown in Table S1 and Table S2). The purified PCR products were cloned into PLB vectors efficiently and conveniently with the Lethal Based Fast Cloning Kit (VT205, Tiangen Biotech, Beijing, China), and the connected products were transformed into *Escherichia coli* DH5*α* competent cells. Then, we screened for plasmid inserts directly from *E. coli* colonies using colony PCR and sent the positive colonies (Figure S2) to Shanghai Sunny Biotech Co. Ltd. for Sanger sequencing. The primers used for colony PCR were as follows: forward—5′-CGACTCACTATAGGGAGAGCGGC-3′ and reverse—5′-TAGGCGTCCTGCAGGCGCT-3′. The PCR conditions are listed in Table S3. The Sanger sequencing primers were as follows: Fseq-1—5′-CGACTCACTATAGGGAGAGCGGC-3′; Fseq-2—5′-CGTGGTCACCCTGTACCACT-3′; Fseq-3—5′-ACTGCTTTCCTGGATTGACCTT-3′; and Rseq—5′-AAGAACATCGATTTTCCATGGCAG-3′.

After confirming the amplified Klotho mRNA sequence by Sanger sequencing, we transplanted it from the PLB-HKL plasmid into the pLVX-CMV-GFP-IRES-Puro vector using AgeI-HF and BamHI-HF to generate the recombinant pLVX-CMV-GFP-HKL-IRES-Puro plasmid (as shown in Figure S1). The pLVX-CMV-GFP-HKL-IRES-Puro plasmid, along with the packaging plasmids pMD2.G and psPAX2, constituted the lentiviral vector system. The lentiviral vector system was transfected into 293 cells to produce the lentivirus. HK-2 cells were infected with this virus and screened for monoclonal cells by treatment with puromycin (2 *μ*g/ml). Then, the mRNA overexpression efficiency was determined, and protein levels were detected by real-time PCR and western blotting (Figure S3). Results demonstrated that the lentiviral vector could infect HK-2 cells and increase the expression of Klotho gene.

### 2.5. Plasmid Construction and Klotho KO in HK-2 Cells

Klotho KO cell lines were generated using the CRISPR/Cas9 system. Briefly, HK-2 cells were cotransfected with LentiCRISPRv2 vectors expressing Cas9 and sgRNA targeting exon 3 of Klotho (Table S4 and Figure S4) as well as a selection gene, such as a puromycin resistance gene. Klotho depletion was accurately monitored in monocolonies by real-time PCR, western blotting, and cell-PCR sequencing (Figures S5–S7). Cell-PCR was performed using the highly efficient and high-fidelity PCR enzyme KOD FX (TOYOBO; KFX-101), and the primers used were as follows: forward—5′-CAGACACAAAGCTGGGTGTCT-3′ (simultaneously as a sequencing primer) and reverse—5′-TCAGCTGACTTACTTGAATGTAGTT-3′. The PCR reaction conditions are listed in Table S5. The results showed that the Klotho gene was successfully knocked out in HK-2 cells.

### 2.6. Effect of Klotho KO and Klotho OE on Inflammation, Fibrosis, and Oxidative Stress in HK-2 Cells

HK-2 cells were divided into 6 groups: normal glucose (NG, 5.5 mM), HG (60 mM), HG + EGCG (1 *μ*M), HG + Klotho KO, HG + Klotho KO + EGCG, and HG + Klotho OE groups. Cells and clear supernatants in each group were harvested at 72 h post treatment, and used for the detection of fibrosis and inflammatory and oxidative stress markers by enzyme-linked immunosorbent assay (ELISA) and western blotting.

### 2.7. ELISA

The expression of inflammatory and oxidative stress markers was detected using ELISA kits. The 8-OHdG ELISA kit (Nanjing Jiancheng Bioengineering Institute, Nanjing, Jiangsu, China) and the 8-iso-prostaglandin F2*α* (8-iso-PGF2*α*) ELISA kit (Cayman Chemical, Ann Arbor, MI, USA) were used to measure mice urine 8-OHdG and 8-iso-PGF2*α*, respectively. The levels of superoxide dismutase (SOD), malondialdehyde (MDA), catalase (CAT), 8-OHdG, interleukin-1*β* (IL-1*β*), interleukin-6 (IL-6), and tumor necrosis factor-*α* (TNF-*α*) in mice kidney tissues and HK-2 cell culture supernatants were measured using commercial kits (Nanjing Jiancheng Bioengineering Institute, Nanjing, Jiangsu, China).

### 2.8. Western Blot Analysis

The protein levels of Klotho, DNA methyltransferase 1 (DNMT1), DNMT3a, DNMT3b, transforming growth factor-*β*1 (TGF-*β*1), and *α*-smooth muscle actin (*α*-SMA) were evaluated by western blot analysis. Primary antibodies against TGF-*β*1 and *α*-SMA were purchased from Santa Cruz Biotechnology, Inc. (Santa Cruz, CA, USA). Primary antibodies against Klotho, DNMT1, DNMT3a, DNMT3b, *β*-actin, and glyceraldehyde-3-phosphate dehydrogenase (GAPDH) and secondary antibodies were obtained from Abcam (Cambridge, MA, USA).

Kidney tissue samples and HK-2 cells were lysed with RIPA Buffer (EpiZyme Inc., Shanghai, China) and centrifuged at 2200 g for 10 min at 4°C. Total protein concentration in the supernatants was determined by a BCA assay (Pierce, Rockford, IL, USA). Around 30 *μ*g of protein was added in each lane and electrophoresed on 10 or 12% sodium dodecyl sulfate-polyacrylamide gels, and then transferred onto PVDF membranes (Bio-Rad, CA, USA). The membranes were blocked with 5% nonfat milk or 5% bovine serum albumin and blocking buffer (1 × Tris − buffered saline and 0.1% Tween 20, pH 7.4) at room temperature (25°C) for 1 h and then incubated with the following primary antibodies overnight at 4°C: Klotho (1 : 1000), DNMT1 (1 : 1000), DNMT3a (1 : 1000), DNMT3b (1 : 1000), TGF-*β*1 (1 : 100), *α*-SMA (1 : 100), *β*-actin (1 : 5000), and GAPDH (1 : 1000). The next day, the membranes were incubated with secondary antibodies (horseradish peroxidase-conjugated anti-rabbit IgG, 1 : 2000) for 1 h at room temperature. Finally, the protein bands were visualized using the SuperSignal West Femto Substrate (Pierce, Rockford, IL, USA).

### 2.9. DNA Pyrosequencing

Genomic DNA from mice kidney tissue and HK-2 cell lines was extracted using the QIAamp DNA Mini Kit (Qiagen, Germantown, MD, USA). DNA concentration and quality were determined using the NanoDrop 2000c Spectrophotometer (Thermo Fisher Scientific, Massachusetts, USA). Next, the DNA samples were subjected to bisulfite treatment with the EZ DNA Methylation-Gold™ Kit (Zymo Research, CA, USA). All procedures were carried out according to the manufacturer's instructions. The CGI region sequence of mice (927 bp, position: chr5:151755140-151756066 (mm9/Mouse)) and the HK-2 cell line (1500 bp, position: chr13:33589850-33591428 (hg19/Human)) was obtained from the University of California Santa Cruz (UCSC) Genome Browser. The transcription start site was +1. A biotin-labeled reverse primer was used for the second round of PCR. Promoter regions were amplified by PCR using Klotho-specific primers. PCR and pyrosequencing primers for the pyrosequencing experiments were generated manually (Tables S6–S9). Average core promoter and site-specific CpG methylation was determined as percentage using the PyroMark Q96 ID System (Qiagen).

### 2.10. ChIP Assay at the Klotho Promoter

A ChIP assay was performed following a protocol used in our lab. HK-2 cells (1.5 × 10^7^) were incubated with 1% formaldehyde for cross-linking at room temperature for 10 min and quenched with 0.125 M for 5 min. Cells were then harvested by scraping and centrifuged at 2,000 g for 1 min. The cell membrane and nuclei were lysed using cell lysis buffer and cell nuclear lysis buffer, respectively. The sonication fragment sizes are typically between 200 bp and 1 kb. The anti-DNMT1 antibody (Abcam; ab13537) and the anti-DNMT3a antibody (Abcam; ab2850) were coated overnight to pull down chromatin. Rabbit IgG served as a negative control. Around 1/10 volume of IP DNA ultrasonic solution was used as the input DNA. After overnight incubation with proteinase K at 58°C for reverse cross-linking, pure DNA was eluted with the MiniElute PCR Purification Kit (28006; Qiagen). The purified DNA was analyzed by real-time PCR for Klotho promoters. Multiple ChIP-qPCR primers were designed for the CpG island of the Klotho promoter. The most effective primer pair was as follows: forward—5′-TTCAGCGCACGGCGAAGTTC-3′ and reverse—5′-TCCTCTGAGAGCAGCC CTG-3′. Relative data quantification was performed using the 2^–ΔΔCt^ method and expressed as fold enrichment.

### 2.11. Statistical Analysis

The Stata 14.0 statistical software (Stata) was used for statistical analyses. Results are expressed as means ± standard error of mean (SEM) from triplicate assays. In a normal distribution, differences among groups were determined using one-way analysis of variance (ANOVA). In a nonnormal distribution, differences between groups were analyzed using the Mann-Whitney *U* test and the Wilcoxon signed-rank test. *P* < 0.05 was considered significantly different.

## 3. Results

### 3.1. Changes in Oxidative Stress Parameters and Inflammatory Cytokine Levels in Mice of Different Groups

Table S10 and Table S11 show the changes in body weight (BW), 24 h urinary protein, oxidative stress parameters, and inflammatory cytokine levels in mice after treatment with or without EGCG by gavage. Compared to those in the normal group, the levels of the 24 h urine proteins, 8-iso-PGF2*α* and 8-OhdG, were increased in the control group. After treatment of db/db mice with EGCG, the levels of urinary 8-iso-PGF2*α* and 8-OHdG were decreased remarkably during the 8-week period (*P* < 0.05). Additionally, EGCG treatment significantly decreased the levels of ROS, MDA, 8-OHdG, IL-1*β*, IL-6, and TNF-*α* in kidney tissue samples compared to those in nontreated db/db mice (*P* < 0.05). Moreover, the level of CAT and SOD was increased in EGCG-treated db/db mice compared with that in nontreated db/db mice (*P* < 0.05).

### 3.2. Changes in the Expression of Klotho, DNMT1, DNMT3a, and DNMT3b in the Kidney Tissues of Mice

**Figure 1:**
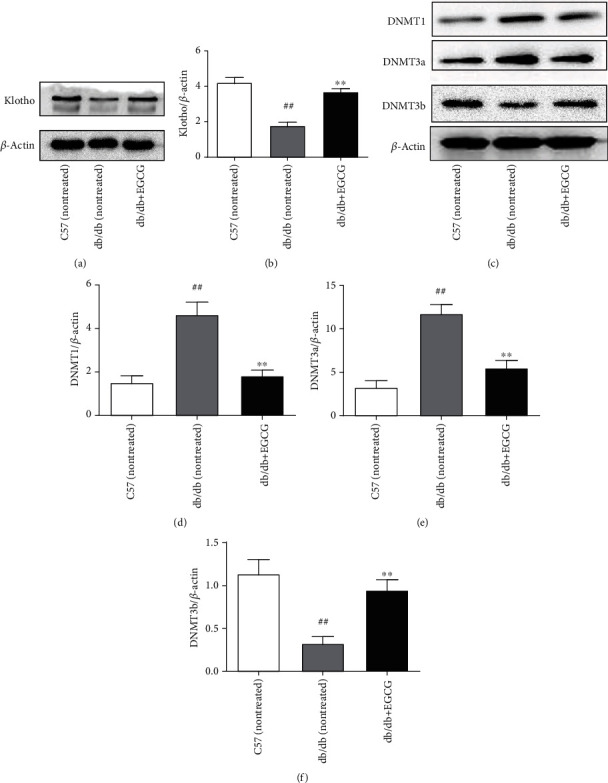
Detection of the protein expression of Klotho, DNMT1, DNMT3a, and DNMT3b in the kidney tissues of mice by western blotting. (a and b) The protein expression of Klotho. (c–e) The protein expression of Klotho, DNMT1, DNMT3a, and DNMT3b. Normal group: nontreated C57 mice. Control group: nontreated db/db mice. EGCG group: db/db mice treated with EGCG (50 mg/kg/d). Values are expressed as means ± SEM. ^∗∗^*P* < 0.01 vs. nontreated db/db mice. ^##^*P* < 0.01 vs. nontreated C57 mice.

The levels of Klotho and DNMT3b were upregulated in EGCG-treated db/db mice compared with those in nontreated db/db mice (Figures 1(a), 1(b), and 1(f); *P* < 0.01). Likewise, compared with that in the control group, the protein expression of DNMT1 and DNMT3a was also markedly downregulated after oral administration of EGCG for 8 weeks (Figures 1(c)–1(e); *P* < 0.01).

### 3.3. Changes in the Klotho Promoter Methylation Level in the Kidney Tissues of Mice

**Figure 2:**
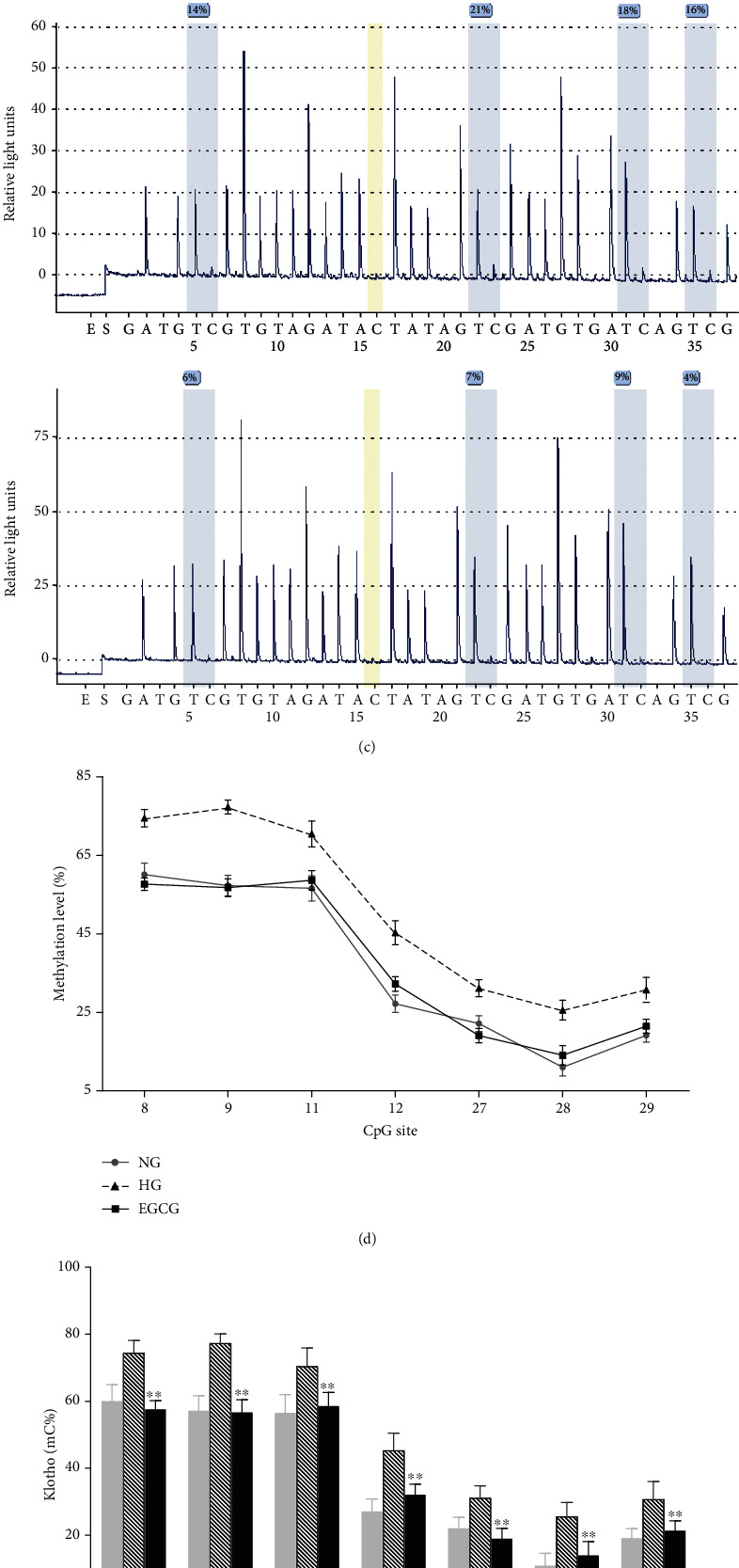
Detection of Klotho promoter methylation level in the kidney tissues of mice and HK-2 cells by pyrosequencing. (a) Significant changes of 14 CpG sites in the Klotho promoter of mice (chr5:151755140-151756066; mm9/mouse). (b) The methylation level of 6 CpG sites in each group of mice. Normal group: nontreated C57 mice. Control group: nontreated db/db mice. EGCG group: db/db mice treated with EGCG (50 mg/kg/d). (c) Representative pyrograms. The upper pyrogram is the normal group, the middle pyrogram is the control group, and the lower pyrogram is the EGCG group. Values are expressed as means ± SEM. ^∗∗^*P* < 0.01 vs. control. ^∗∗^*P* < 0.01 vs. nontreated db/db mice. ^##^*P* < 0.01 vs. nontreated C57 mice. (d) Significant changes in 7 CpG sites in the Klotho promoter of HK-2 cells (chr13: 33589850-33591428; hg19/human). (e) The methylation level of the Klotho promoter in each group of HK-2 cells. NG: normal glucose (5.5 mM); HG: high glucose (60 mM); EGCG: HK-2 cells treated with 1 *μ*M EGCG. (f) Representative pyrograms. The upper pyrogram is the NG group, the middle pyrogram is the HG group, and the lower pyrogram is the EGCG group. Values are expressed as means ± SEM. ^∗∗^*P* < 0.01 vs. the HG group.

Site-specific methylation was detected in 94 out of 101 CpG sites in the Klotho promoter by pyrosequencing. Among them, there were significant changes in the 10th, 13th, 14th, 44th, 56th, 62th, 65th, 79th, 88th, 89th, 90th, 91th, and 99th CpG sites. Compared with that in the normal group, the methylation level of the Klotho promoter was significantly increased in nontreated db/db mice (control group), while EGCG treatment for 8 weeks significantly reduced the methylation level of the Klotho promoter in db/db mice compared with that in nontreated db/db mice (Figures 2(a)–2(c); *P* < 0.01).

### 3.4. Effects of EGCG on HG-Induced Injury in HK-2 Cells

**Figure 3:**
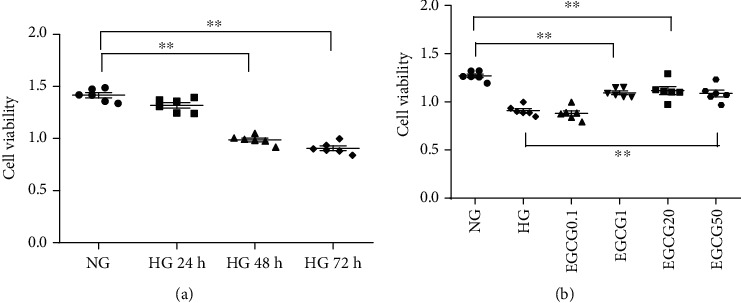
Effect of EGCG on HK-2 cell viability under HG conditions. (a) Effect of HG on HK-2 cell viability at different time points. ^∗∗^*P* < 0.01 vs. the HG group at 24 h. (b) Effect of HG on the viability of HK-2 cells treated with EGCG for 72 h. ^∗∗^*P* < 0.01 vs. the HG group. HG: high glucose (60 mM). Values are expressed as means ± SEM.

We first detected the effect of HG (60 mM) on the activity of HK-2 cells, and then investigated whether this effect was time-dependent. Compared to NG treatment (5.5 mM), treatment with HG for 24 h did not induce any significant changes in cell viability. However, we found that HK-2 cell activity was significantly decreased under HG conditions in a time-dependent manner (Figure 3(a); *P* < 0.01). Next, HK-2 cells were treated with increasing doses of EGCG in HG conditions, and cell viability was assessed. Compared to HG treatment alone, additional treatment with EGCG (doses of 1, 20, and 50 *μ*M) for 72 h significantly increased cell viability (Figure 3(b); *P* < 0.01).

**Figure 4:**
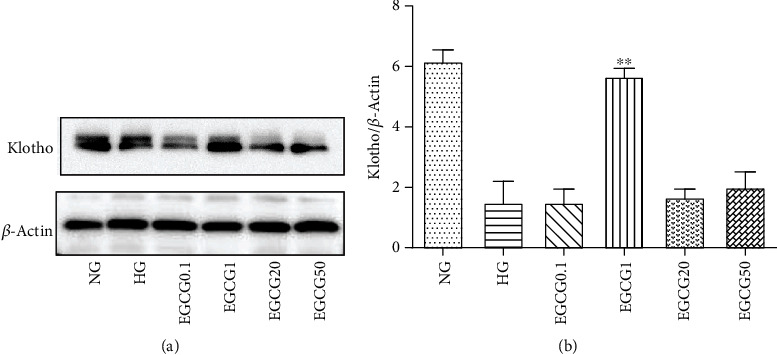
The protein expression of Klotho in HK-2 cells detected by western blotting. (a) Effect of different doses of EGCG on Klotho expression in HG-induced HK-2 cells. (b) The expression of Klotho/*β*-actin was quantitatively analyzed in each group. NG: normal glucose (5.5 mM); HG: high glucose (60 mM); EGCG0.1, EGCG1, EGCG20, and EGCG50: HK-2 cells treated with EGCG doses of 0.1, 1, 20, and 50 *μ*M in high-glucose conditions, respectively. Values are expressed as means ± SEM. ^∗∗^*P* < 0.01 vs. the HG group.

Furthermore, we examined the changes in Klotho expression in HK-2 cells. The protein expression of Klotho was upregulated in the HG + EGCG group compared to that in the HG group. The concentration of 1 *μ*M EGCG was used for all subsequent experiments because Klotho protein expression was significantly increased at this concentration (Figure 4; *P* < 0.05).

### 3.5. Changes in the Expression of Klotho, DNMT1, DNMT3a, and DNMT3b in HK-2 Cells

**Figure 5:**
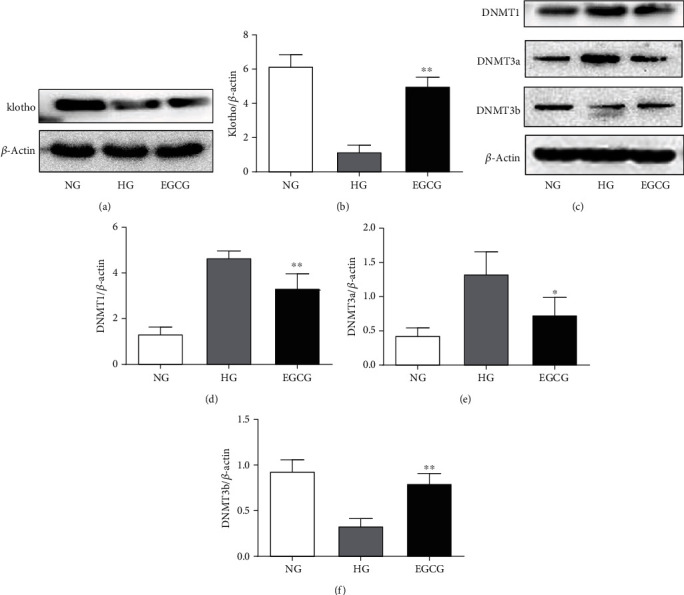
The protein expression of Klotho, DNMT1, DNMT3a, and DNMT3b in HK-2 cells detected by western blotting. (a and b) The protein expression of Klotho. (c–e) The protein expression of Klotho, DNMT1, DNMT3a, and DNMT3b. NG: normal glucose (5.5 mM); HG: high glucose (60 mM); EGCG: HK-2 cells treated with 1 *μ*M EGCG. Values are expressed as means ± SEM. ^∗^*P* < 0.05 vs. the HG group; ^∗∗^*P* < 0.01 vs. the HG group.

As shown in Figure 5, after EGCG intervention, the HG-induced decrease in Klotho and DNMT3b expression was markedly attenuated (*P* < 0.01). Moreover, we also found that the protein expression of DNMT1 and DNMT3a was obviously increased in the HG group compared to that in the NG group, but significantly decreased after treatment with EGCG for 72 h (Figures 5(c)–5(e); *P* < 0.01).

### 3.6. Changes in the Methylation Level of the Klotho Promoter in HK-2 Cells

Next, site-specific methylation of the Klotho promoter in HK-2 cells was analyzed. Compared with that in the NG group, the methylation level of the Klotho promoter was significantly increased in the HG group, but significantly reduced after EGCG treatment for 72 h (Figures 2(d)–2(f); *P* < 0.01). We detected site-specific methylation in 166 out of 177 CpG sites in the Klotho promoter. Among them, there were significant changes in the 8th, 9th, 11th, 12th, 27th, 28th, and 29th CpG sites.

### 3.7. EGCG Regulated the Enrichment of DNMTs in the Klotho Promoter in HK-2 Cells

**Figure 6:**
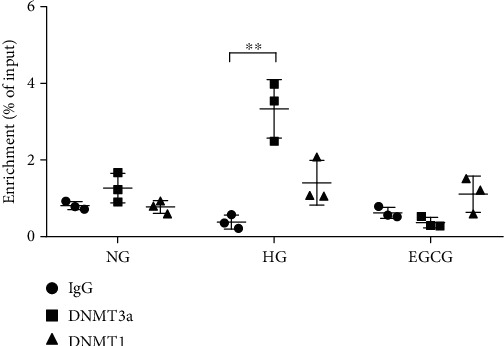
Changes in DNMT1 and DNMT3a binding at the Klotho promoter in HK-2 cells. Relative ChIP enrichment was quantified by qPCR. IgG (immunoglobulin G) was used as the negative control. NG: normal glucose (5.5 mM); HG: high glucose (60 mM); EGCG: HK-2 cells treated with 1 *μ*M EGCG. Values are expressed as means ± SEM. ^∗∗^*P* < 0.01 vs. the HG group.

To determine the underlying mechanism of EGCG in regulating the methylation level of the Klotho gene promoter, we investigated the enrichment of DNMTs in the Klotho promoter in HK-2 cells by ChIP assay. Compared to the NG group, there was an increase in the enrichment of DNMT3a in the Klotho gene promoter in the HG group. After EGCG treatment, the enrichment of DNMT3a in the Klotho promoter was decreased. However, the enrichment of DNMT1 in the Klotho gene promoter was not significantly different between the HG and NG groups, as well as between the HG and EGCG groups (Figure 6; *P* < 0.01).

### 3.8. Effects of HG-Induced Injury on HK-2 Cells with Klotho KO or OE

As shown in Table S12, the HG-induced increase in ROS, TNF-*α*, IL-6, and IL-1*β* expression and decrease in SOD and MDA expression were significantly decreased and increased, respectively (*P* < 0.05), after EGCG intervention. We also found that Klotho OE attenuated HG-induced oxidative stress and inflammation. Meanwhile, the expression of ROS, TNF-*α*, IL-6, and IL-1*β* was increased in Klotho KO HK-2 cells under HG conditions. Moreover, no significant differences in ROS, SOD, MDA, TNF-*α*, IL-6, and IL-1*β* expression levels were observed between Klotho KO HK-2 cells with and without EGCG treatment in HG conditions.

**Figure 7:**
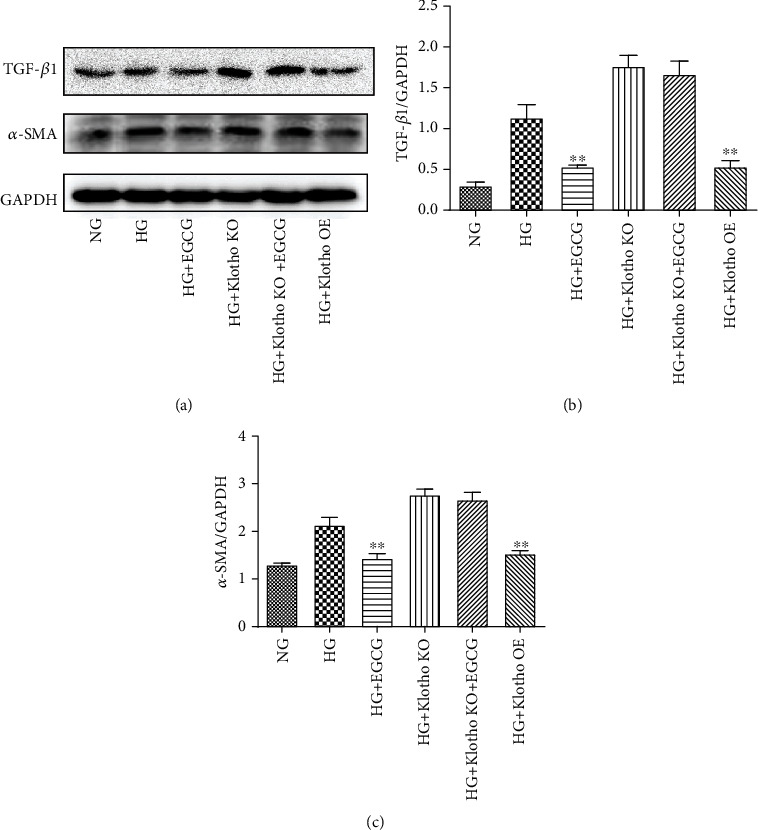
The protein expression of TGF-*β*1 and *α*-SMA in HK-2 cells detected by western blotting. (a) The protein expression of TGF-*β*1 and *α*-SMA in HK-2 cells of each group. The expression of TGF-*β*1/GAPDH (b) and *α*-SMA/GAPDH (c) was quantitatively analyzed in each group. NG: normal glucose (5.5 mM); HG: high glucose (60 mM); EGCG: HK-2 cells treated with 1 *μ*M EGCG; Klotho KO: Klotho gene knockout; Klotho OE: Klotho gene overexpression. Values are expressed as means ± SEM. ^∗∗^*P* < 0.01 vs. the HG group.

As shown in Figure 7, compared to HG treatment alone, the protein expression of TGF-*β*1 and *α*-SMA in HK-2 cells was downregulated by EGCG treatment and Klotho OE. However, the protein level of TGF-*β*1 and *α*-SMA showed no obvious change in Klotho KO HK-2 cells with or without EGCG treatment under HG conditions. Thus, these results indicate that Klotho functions as a key target of EGCG and mediates its anti-inflammatory, antioxidative, and antifibrotic effects.

## 4. Discussion

In this study, we found that treatment with EGCG (50 mg/kg/d) for 8 weeks decreased the level of 24 h urinary proteins, 8-OHdG and 8-iso-PGF2*α*, and also decreased the expression of renal oxidative stress and inflammatory and fibrosis markers in db/db mice. Moreover, renal DNMT1 and DNMT3a levels were increased, the methylation level of the Klotho gene promoter was increased, and the expression of the Klotho protein was reduced in db/db mice. However, these effects could be reversed with EGCG treatment, suggesting that EGCG downregulated the expression of DNMTs, thereby regulating Klotho gene promoter methylation to increase the expression of the Klotho protein. Similar results were obtained in HK-2 cells. The expression of oxidative stress and inflammatory and fibrosis markers was significantly reduced by Klotho OE under HG conditions, indicating that OE of the Klotho protein can alleviate HG-induced cell damage. In the case of Klotho gene KO, the expression of oxidative stress and inflammatory and fibrosis markers was significantly upregulated by HG stimulation, and EGCG treatment did not reverse this effect, indicating that EGCG did not play a role in mediating the effects of the Klotho gene KO. Hence, Klotho was identified to be a key target of EGCG.

In China, tea has a long history and is widely consumed by the general population. EGCG, an extract of green tea, has the advantages of being natural and safe, without any side effects [19]. As reported previously, EGCG has many functions, such as anti-inflammatory, antioxidative, and antifibrotic effects in various tissues and cells, and it has been shown to inhibit DNA methylation in tumor cell lines [20–23]. However, the underlying molecular mechanism by which EGCG exerts these protective effects remains unknown. Some studies have shown that EGCG plays an important role in kidney protection by exerting multiple functions, but the exact mechanism is not clear.

Recent studies have shown that Klotho plays an important role in the development of kidney disease [24–26]. The protein expression of Klotho was found to be decreased in kidney tissues in several clinical and animal studies on kidney disease, including DKD [27, 28]. It has been reported that the decrease of plasma Klotho is closely related to the progression of DKD in patients with type 2 diabetes [29]. Previous studies have shown that renal Klotho protein was downregulated in STZ-induced diabetic mice [30]. Kadoya et al. [31] showed that Klotho could alleviate glomerular hypertrophy and glomerular injury in DKD. Similar results were obtained in our study. The protein expression of Klotho was decreased in db/db mice and HK-2 cells under HG conditions.

Epigenetics refers to the ability to regulate gene expression without altering the primary nucleotide sequence, i.e., phenotypic changes, including histone modifications, DNA methylation, and noncoding RNAs can be induced without changing the genotype [32, 33]. In general, epigenetic modifications are thought to be stable and heritable during cell division [34]. However, they may be reversible and affected by environmental factors (oxidative stress, inflammation, cytokines, growth factors, and so on), age, and disease state [4, 5]. Studies have shown that epigenetic mechanisms, especially DNA methylation, can lead to various kidney diseases, including DKD, chronic kidney disease, and renal cell carcinoma [35]. Recent studies have suggested that DNA methylation is closely related to the pathogenesis and progression of DKD [36, 37].

In our study, Klotho and DNMT3b protein expression was upregulated, while DNMT1 and DNMT3a protein expression was downregulated in the EGCG group compared with that in the control group (db/db mice without EGCG treatment). The methylation level of the Klotho gene promoter was increased in the control group but decreased in the EGCG group. Similar to our results, Zhang et al. found that DNMT3b protein expression was decreased and DNMT3a protein expression was increased in chronic nondiabetic kidney disease [38]. Moreover, in previous studies, the protein expression of both DNMT3a and DNMT3b was found to be increased in tumor cell lines [39]. Hence, the protein expression of DNMT3a and DNMT3b may be associated with the development of different diseases, and further research is required to study the differences in DNMT3a expression and DNMT3b expression in different diseases or cells.

Oxidative stress, inflammatory response, and fibrosis are the major pathological mechanisms of DKD. ROS are mainly produced by excessive oxidative stress, and renal tubular cells are the main target cells of ROS. ROS can induce the apoptosis of mesangial cells and tubular cells, causing pathological changes, which ultimately lead to glomerular sclerosis [40, 41]. Inflammatory response is the major cause of DKD [42]. In addition, inflammatory response can also induce oxidative stress under HG conditions, and vice versa, forming a vicious circle [43]. It has been reported that oxidative stress can increase the expression of TGF-*β*1 and *α*-SMA in DKD in a direct or indirect manner [44]. Both TGF-*β*1 and *α*-SMA are hypertrophic and fibrotic cytokines that play an important role in glomerular hypertrophy and mesangial matrix broadening, which ultimately lead to end-stage renal disease [45]. In this study, our data demonstrated that both EGCG and Klotho OE could decrease the levels of oxidative stress and inflammatory and fibrosis markers.

Next, we investigated the mechanism by which EGCG regulates Klotho expression to protect the kidney in DKD. Klotho is highly expressed in the distal tubules of the kidney and is rich in CpG islands, indicating that the promoter of the Klotho gene is closely related to DNA methylation. Therefore, we examined the methylation level of the Klotho gene promoter and found that its level was increased and the expression of Klotho protein was decreased in db/db mice compared with that in normal mice. Similar results were obtained in HG-induced HK-2 cells. These findings indicated that changes in Klotho promoter methylation were observed in DKD. After 8 weeks of intervention with EGCG (50 mg/kg/d), results showed that the methylation level of the Klotho gene promoter in db/db mice was significantly lower than that in control mice, indicating that EGCG can reverse Klotho promoter methylation. We also found that EGCG could regulate the expression of DNMTs in db/db mice and HK-2 cells. Moreover, ChIP results showed that DNMT3a and Klotho promoters were highly enriched in HG environment, and the enrichment was reduced after EGCG intervention. The effect of DNMT3a on Klotho promoter methylation has been reported in several studies [37, 46, 47]; however, the binding of DNMT3a to Klotho gene promoters was not reported. In this study, for the first time, we showed that EGCG decreased the enrichment of DNMT3a in the Klotho gene promoter under HG conditions.

Hence, these results showed that HG increased the methylation level of the Klotho gene promoter by regulating DNMTs, leading to low expression of Klotho protein and causing changes in downstream molecules, eventually upregulating the expression of oxidative stress and inflammatory and fibrosis markers, and thus accelerating the progression of DKD. Based on our results, EGCG could downregulate the enrichment of DNMT3a and Klotho to decrease the methylation level of the Klotho gene promoter, and it could also upregulate the expression of Klotho to inhibit oxidative stress, fibrosis, and inflammation. In HG-induced HK-2 cells, Klotho OE could inhibit the expression of oxidative stress and inflammatory and fibrosis markers, and ameliorate cell damage. However, in the case of Klotho gene KO, EGCG intervention did not significantly decrease marker expression. These results indicate that EGCG does not exert its protective function in the absence of the Klotho gene.

### 4.1. Limitation

In animal experiments, we have three groups. C57BLKS/J normal mice were set as blank controls, C57BLKS/J db/db mice were set as negative controls, and C57BLKS/J db/db mice treated with EGCG at 50 mg/kg/d were set as the experimental group. The C57BLKS/J normal mice treated with only EGCG were missing. If C57BLKS/J normal mice treated with only EGCG were set, the experiment would be more rigorous.

## 5. Conclusion

**Figure 8:**
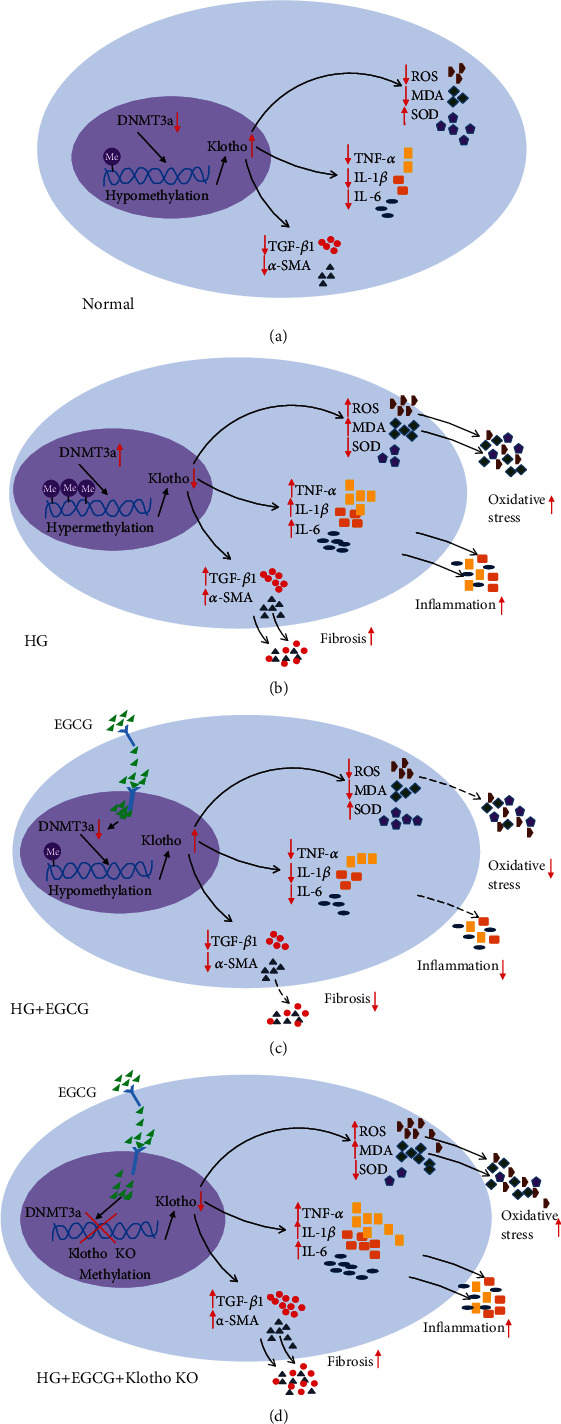
Schematic representation of the underlying mechanisms by which EGCG regulates HG-induced oxidative stress, inflammation, and renal fibrosis. (a–d) The expression of oxidative stress, inflammation, and renal fibrosis markers under normal, HG, HG+EGCG, and HG+EGCG+Klotho KO conditions.

In summary, our results suggested that HG treatment increased the methylation level of the Klotho gene promoter via regulating DNMT3a expression (Figures 8(a) and 8(b)), while EGCG exerted renoprotective effects through its demethylation function and decreased Klotho gene promoter methylation in HG conditions by inhibiting the expression of fibrosis, oxidative stress, and inflammatory markers (Figure 8(c)). We also found that the expression of fibrosis, oxidative stress, and inflammatory markers was increased in Klotho KO HK-2 cells treated with EGCG under HG conditions (Figure 8(d)). Hence, our study suggests that Klotho is a key target of EGCG through which it exerts its protective functions.

## Data Availability

The datasets generated and/or analyzed during the current study are available from the corresponding authors upon reasonable request.

## Supplementary Materials

Supplementary Materials Table S1: Klotho cDNA PCR primers. Table S2: sequence of PCR product. Figure S1: Klotho OE plasmid map. Table S3: Colony PCR using the thermal cycling program. Figure S2: Klotho OE plasmid inserts were identified by colony PCR. M: 100 bp DNA marker; lanes 1-7: *E. coli* colonies 1-7. Figure S3: the identification of Klotho OE in HK-2 cells. Table S4: the oligo sequence of HKL-KO. Figure S4: Klotho KO plasmid map. Table S5: cell-PCR using the thermal cycling program. Figure S5: the determination of Klotho knockout efficiency by PCR. Figure S6: the determination of Klotho knockout efficiency by western blot. Figure S7: monoclonal cell-PCR sequencing alignment results and sequencing quality check peak map. Sequencing showed double peaks and gene sequence changes. Table S6: nested PCR for mice. Table S7: pyrosequencing primers and detection sequence of mice. Table S8: nested PCR for HK-2 cells. Table S9: pyrosequencing primers and detection sequence of HK-2 cells. Table S10: changes in body weight, 24-hour urinary protein, urinary 8-iso-PGF2*α*, and 8-OHdG in mice after different treatments. Table S11: changes in parameters of oxidative stress and proinflammatory cytokines in kidney homogenates in mice after different treatments. Table S12: changes in parameters of oxidative stress and proinflammatory cytokines in HK-2 cells after different treatments. Figure S8: a graphical illustration of the mouse treatment plan.
