# Supplementary material for: EGCG Attenuates Renal Damage via Reversing Klotho Hypermethylation in Diabetic db/db Mice and HK-2 Cells
Source: Oxid Med Cell Longev. 2020 Aug 27;2020:6092715. doi: 10.1155/2020/6092715 (PMC7474393; doi:10.1155/2020/6092715)
Supplement: Supplementary Materials — Table S1: Klotho cDNA PCR primers. Table S2: sequence of PCR product. Figure S1: Klotho OE plasmid map. Table S3: Colony PCR using the thermal cycling program. Figure S2: Klotho OE plasmid inserts were identified by colony PCR. M: 100 bp DNA marker; lanes 1-7: E. coli colonies 1-7. Figure S3: the identification of Klotho OE in HK-2 cells. Table S4: the oligo sequence of HKL-KO. Figure S4: Klotho KO plasmid map. Table S5: cell-PCR using the thermal cycling program. Figure S5: the determination of Klotho knockout efficiency by PCR. Figure S6: the determination of Klotho knockout efficiency by western blot. Figure S7: monoclonal cell-PCR sequencing alignment results and sequencing quality check peak map. Sequencing showed double peaks and gene sequence changes. Table S6: nested PCR for mice. Table S7: pyrosequencing primers and detection sequence of mice. Table S8: nested PCR for HK-2 cells. Table S9: pyrosequencing primers and detection sequence of HK-2 cells. Table S10: changes in body weight, 24-hour urinary protein, urinary 8-iso-PGF2α, and 8-OHdG in mice after different treatments. Table S11: changes in parameters of oxidative stress and proinflammatory cytokines in kidney homogenates in mice after different treatments. Table S12: changes in parameters of oxidative stress and proinflammatory cytokines in HK-2 cells after different treatments. Figure S8: a graphical illustration of the mouse treatment plan. [file 6092715.f1.docx]

TableS1: klotho cDNA PCR primers

| Name | Sequence (5’-3’) |
| --- | --- |
| F-HKL-AgeI | GGGACCGGTATGCCCGCCAGCGC |
| R-HKL-BamHI | CGGGATCCCTAGTGATGATGATGATGATGGTGATAGGGCTTGGTGAGACT |

Note: F, forward; R, reverse; AgeI digestion sequence：ACCGGT; BamhI digestion sequence：GGATCC

TableS2: Sequence of PCR product (length: 1685bp)

| gggaccggtatgcccgccagcgccccgccgcgccgcccgcggccgccgccgccgtcgctgtcgctgctgctggtgctgctgggcctgggcggccgccgcctgcgtgcggagccgggcgacggcgcgcagacctgggcccgtttctcgcggcctcctgcccccgaggccgcgggcctcttccagggcaccttccccgacggcttcctctgggccgtgggcagcgccgcctaccagaccgagggcggctggcagcagcacggcaagggtgcgtccatctgggatacgttcacccaccaccccctggcacccccgggagactcccggaacgccagtctgccgttgggcgccccgtcgccgctgcagcccgccaccggggacgtagccagcgacagctacaacaacgtcttccgcgacacggaggcgctgcgcgagctcggggtcactcactaccgcttctccatctcgtgggcgcgagtgctccccaatggcagcgcgggcgtccccaaccgcgaggggctgcgctactaccggcgcctgctggagcggctgcgggagctgggcgtgcagcccgtggtcaccctgtaccactgggacctgccccagcgcctgcaggacgcctacggcggctgggccaaccgcgccctggccgaccacttcagggattacgcggagctctgcttccgccacttcggcggtcaggtcaagtactggatcaccatcgacaacccctacgtggtggcctggcacggctacgccaccgggcgcctggcccccggcatccggggcagcccgcggctcgggtacctggtggcgcacaacctcctcctggctcatgccaaagtctggcatctctacaatacttctttccgtcccactcagggaggtcaggtgtccattgccctaagctctcactggatcaatcctcgaagaatgaccgaccacagcatcaaagaatgtcaaaaatctctggactttgtactaggttggtttgccaaacccgtatttattgatggtgactatcccgagagcatgaagaataacctttcatctattctgcctgattttactgaatctgagaaaaagttcatcaaaggaactgctgacttttttgctctttgctttggacccaccttgagttttcaacttttggaccctcacatgaagttccgccaattggaatctcccaacctgaggcaactgctttcctggattgaccttgaatttaaccatcctcaaatatttattgtggaaaatggctggtttgtctcagggaccaccaagagagatgatgccaaatatatgtattacctcaaaaagttcatcatggaaaccttaaaagccatcaagctggatggggtggatgtcatcgggtataccgcatggtccctcatggatggtttcgagtggcacagaggttacagcatcaggcgtggactcttctatgttgactttctaagccaggacaagatgttgttgccaaagtcttcagccttgttctaccaaaagctgatagagaaaaatggcttccctcctttacctgaaaatcagcccctagaagggacatttccctgtgactttgcttggggagttgttgacaactacattcaagtaagtcagctgacaaaaccaatcagcagtctcaccaagccctatcaccatcatcatcatcatcactagggatcccg |
| --- |


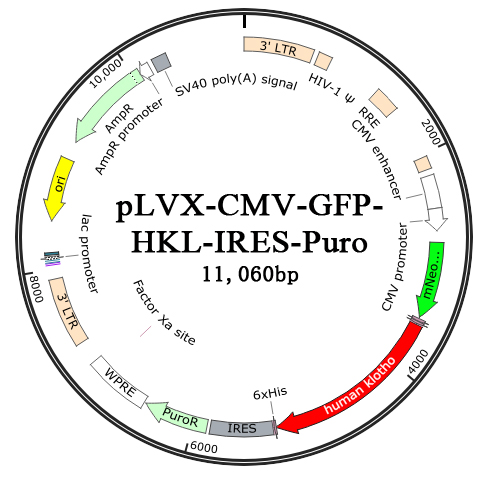


FigureS1: klotho OE plasmid map

TableS3：colony PCR using the thermal cycling program below

| Step | Temperature (℃) | Time |
| --- | --- | --- |
| (1) Denature | 95 | 2 min |
| (2) Denature | 98 | 10 s |
| (3) Anneal | 58 | 30 s |
| (4) Extend | 72 | 1 min |
| Repeat steps 2–4 for 30-35 cycles |  |  |
| (5) Extend | 72 | 5 min |
| (6) Hold | 4 | Indefinite |


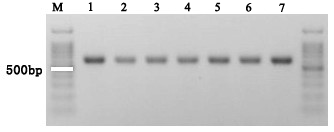


FigureS2: klotho OE inserts plasmid were identified by Colony PCR

M: 100bp DNA marker; Lane1-7: E. coli colony 1-7


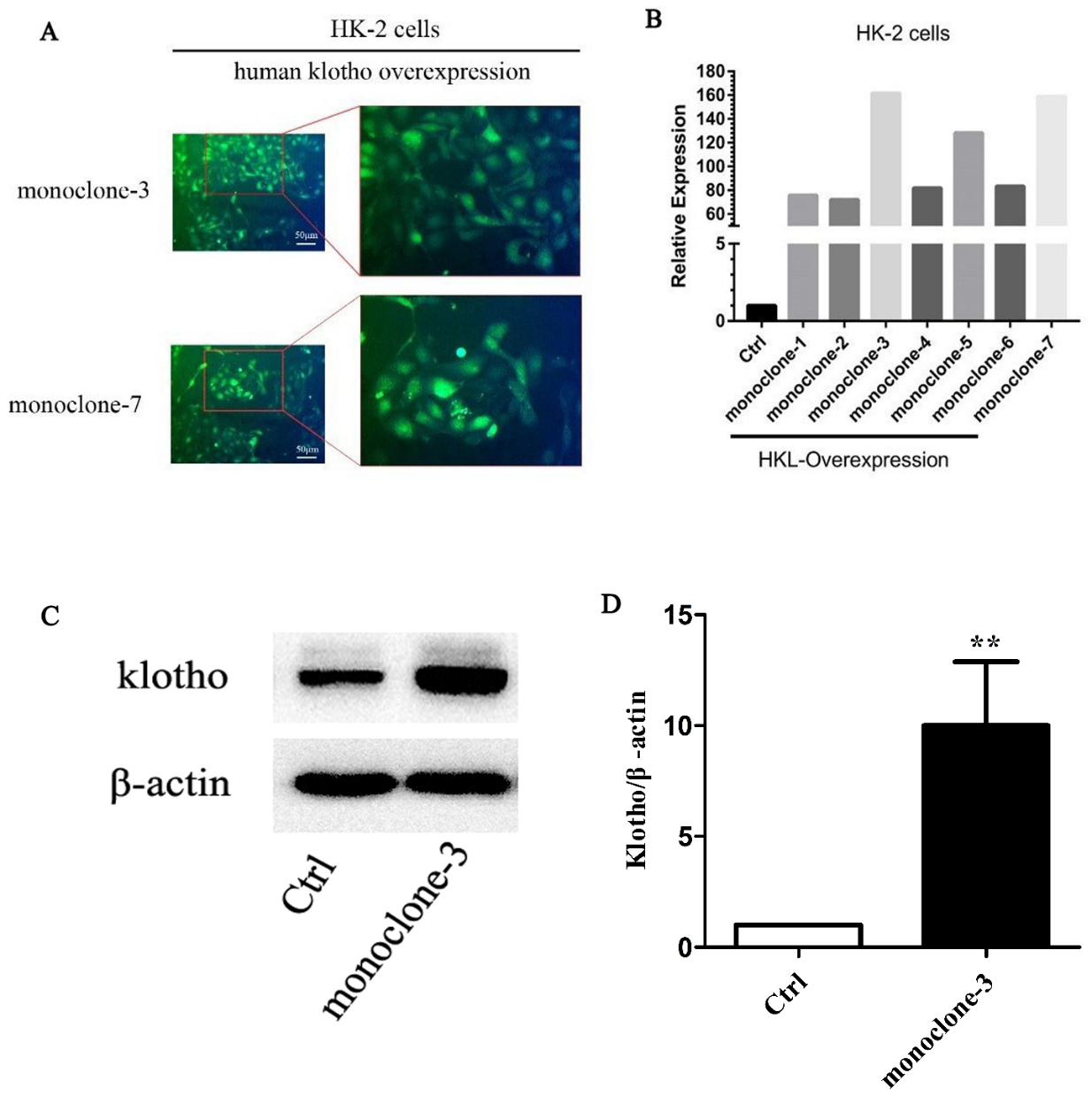


FigureS3: The identification of klotho OE in HK-2 cells

The GFP expression were evaluated by fluorescence microscopy (A). The mRNA expression of klotho by klotho OE in HK-2 cells detected by PCR (B). The protein level of klotho by klotho OE in HK-2 cells detected by western blot (C and D). Scale bar: 50 μm; Ctrl: control group (klotho empty plasmid); monoclone1-7: klotho OE plasmid. Monoclone-3 was used in future study.

TableS4: The Oligo sequence of HKL-ko

| HKL-ko-Oligo1 | CACCGGTATACCGCATGGTCCCTCA |
| --- | --- |
| HKL-ko-Oligo2 | AAACTGAGGGACCATGCGGTATACC |


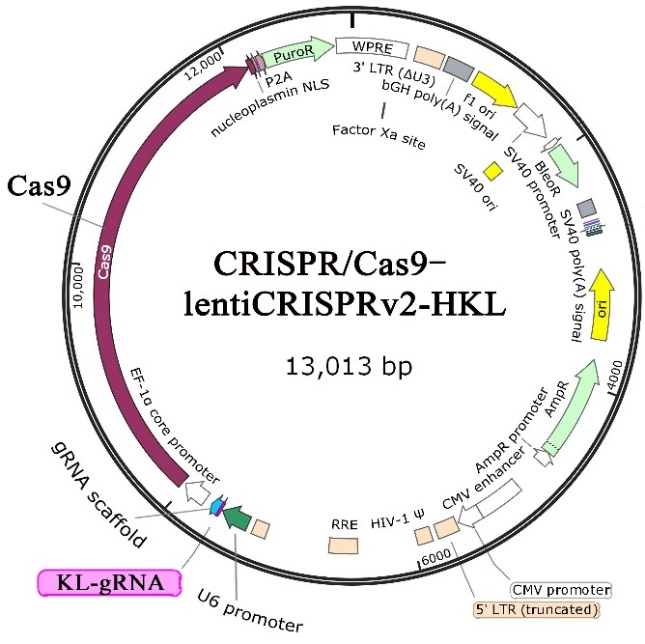


FigureS4: klotho KO plasmid map

TableS5：Cell- PCR using the thermal cycling program below

| Step | Temperature (℃) | Time |
| --- | --- | --- |
| (1) Denature | 94 | 5 min |
| (2) Denature | 98 | 15 s |
| (3) Anneal | 58 | 30 s |
| (4) Extend | 68 | 1 min |
| Repeat steps 2–4 for 30-35 cycles |  |  |
| (5) Extend | 68 | 5 min |
| (6) Hold | 4 | Indefinite |


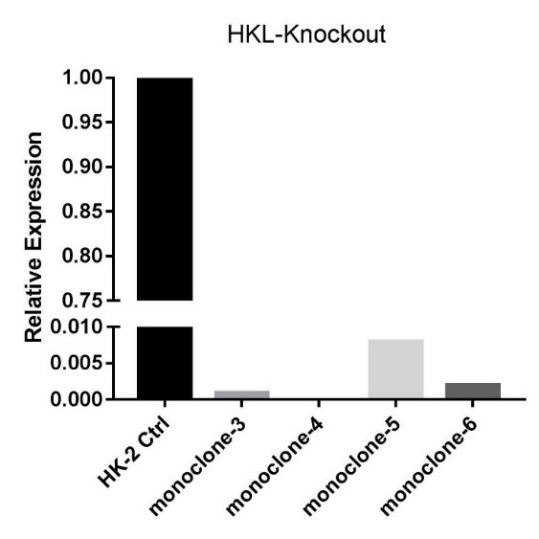


FigureS5: The determination of klotho knockout efficiency by PCR


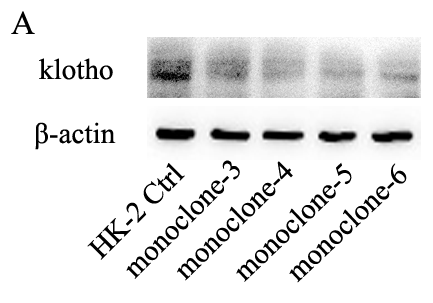

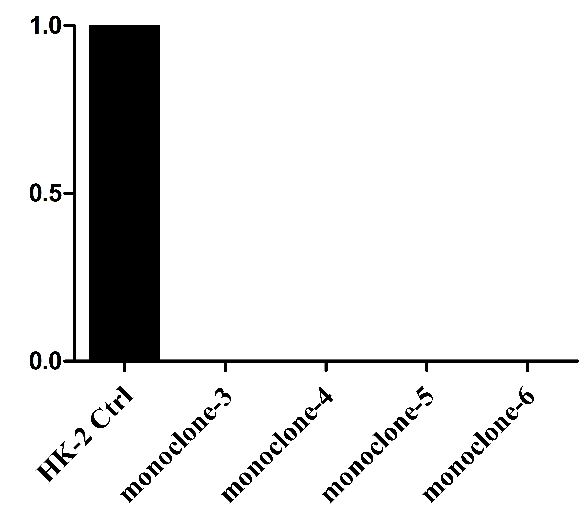


FigureS6: The determination of klotho knockout efficiency by western blot


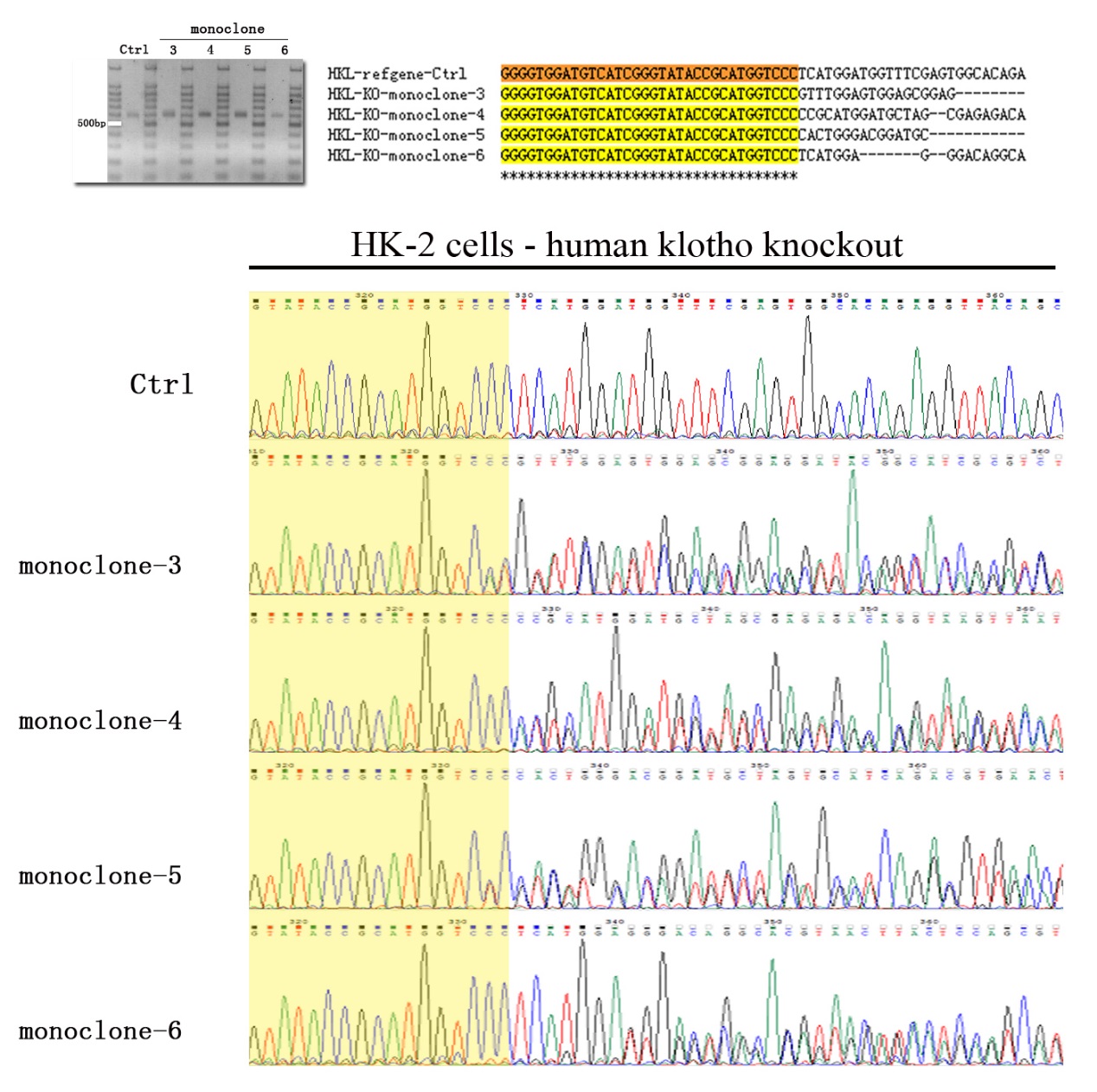


FigureS7: Monoclonal cell PCR sequencing alignment results and sequencing quality check peak map. Sequencing showed double peaks and gene sequence changes.

TableS6: Nested PCR for mice

1. PCR primers

| Name | Sequence | Amplified fragments（bp） |
| --- | --- | --- |
| mKL-CGI-F1.1 | TTATAGGATTGTGYGATGTGGAATAGTT |  |
| mKL-CGI-F1.2 | TTGAGTTGGTTGTAGTAGGTGTTTGTT |  |
| mKL-CGI-R1 | AACCTTCAACACCCCAACCATATACTAACCCRACTCAACRCTCAAACAA | -134---+227 |
| mKL-CGI-F2.1 | GYGGYGTTTGTTTTGGATAATTATTGTT |  |
| mKL-CGI-F2.2 | GGTGTTGTTTYGTTTGTTGTTGTTGTATT |  |
| mKL-CGI-R2 | AACCTTCAACACCCCAACCATATAATTATAACTATCRCTAACCACATCTCCA | +149---+506 |
| mKL-CGI-F3.1 | TTATTGGAGATGTGGTTAGYGATAGTTAT |  |
| mKL-CGI-F3.2 | TTGGAGATGTGGTTAGYG ATAGTTATAAT |  |
| mKL-CGI-R3 | AACCTTCAACACCCCAACCATATATTATAAACAACCAAATACCCRAACCTAA | +478---+925 |
| mKL-CGI-R-Biotin | TATATGGTTGGGGTGTTGAAGGTT |  |

(2) PCR：

First round PCR

| System | Components | Volume |
| --- | --- | --- |
| 10μl | 2*Taq（No.PT102-01, LifeFeng） | 5μl |
|  | F（10μM） | 0.1μl |
|  | R（10μM） | 0.1μl |
|  | Temp | 1.5μl |
|  | ddH_2_O | 3.3μl |
| PCR parameters | 98°1min30s；98°15s，58°30s，72°30s🡪（35cycles）；72°5min；4°+∞ | |

Second round PCR

| System | Components | Volume |
| --- | --- | --- |
| 30μl | 2*Taq | 15μl |
|  | F (10μM) | 0.5μl |
|  | mKl-CGI-R2-Biotin（10μM） | 0.5μl |
|  | Temp（the products of first round PCR） | 4μl |
|  | ddH_2_O | 10μl |
| PCR parameters | 98°1min30s；98°15s，58°30s，72°30s🡪（35cycles）；72°5min；4°+∞ | |

TableS7: Pyrosequencing primers and detection sequence of mice

| Name | Sequence | CpG site# |
| --- | --- | --- |
| mKL-CGI-Fseq-1 | GGTATTTTGGTTGGTTGAGTTTTTGTTT |  |
| detected sequence A  （-42—+31） | *YGTTTTYGTGTTAGGTYGGAGTGGGGGGYGGYGTTTGTTTTGGATAATTATTGTTYGTGGGGYGGYGGGAGYG* | 1-9 |
| mKL-CGI-Fseq-2 | GYGGYGTTTGTTTTGGATAATTATTGTT |  |
| detected sequence B  （+14—+88） | *YGTGGGGYGGYGGGAGYGGGGGTGGGTATYGYGTAGGGAGGGYGGYGGGGYGYGGGTATATAGGGGYGYGGYGYG* | 6-19 |
| mKL-CGI-Fseq-3 | GGTGTTTTTTYGGTTTTYGTAGTATGTTA |  |
| detected sequence C  （+117—+189） | *GTTYGYGTTTTTTTTYGTYGTTYGTYGYGGTTGGTGTTGTTTYGTTTGTTGTTGTTGTATTTGTTGTTGTTYG* | 22-30 |
| mKL-CGI-Fseq-4 | TATTTGTTGTTGTTYGTTTTGYGYGTT |  |
| detected sequence D  （+201—+258） | *YGTTGTTTGAGYGTTGAGTYGGGTTAGGGYGYGTAGATTTGGGTTYGTTTYGYGYGYG* | 33-42 |
| mKL-CGI-Fseq-5 | GTTTYGYGYGYGTTTTTGTTTTAGAGGT |  |
| detected sequence E  （+275—+351） | *YGTTGGTTTTTTTTAYGATATTTTTTTYGAYGGTTTTTTTTGGGYGGTAGGTAGYGTYGTTTATTAGATYGAGGGYG* | 43-51 |
| mKL-CGI-Fseq-6 | GTYGTTTATTAGATYGAGGGYGGTT |  |
| detected sequence F  （+355—+423） | *GGYGATAGTAYGGTAAAGGYGYGTTTATTTGGGATATTTTTATTTATTATTTTGGGGYGGTTTYGTTYG* | 52-58 |
| mKL-CGI-Fseq-7 | ATTATTTTGGGGYGGTTTYGTTYGATT |  |
| detected sequence G  （+427—+495） | *TTTYGATYGTYGTGGYGTYGTYGGGTGTTTYGTYGTTTTTTTTGTTTTTTATTGGAGATGTGGTTAGYG* | 59-67 |
| mKL-CGI-Fseq-8 | GAGATGTGGTTAGYGATAGTTATAATAA |  |
| detected sequence H  （+509—+580） | *YGTTTATYGYGATATAGAGGGGTTGYGYGAATTGGGGGTTATTTATTATYGTTTTTTTATATYGTGGGYGYG* | 68-76 |
| mKL-CGI-Fseq-9 | TGGGYGYGGGTGTTTTTTAATGGTAT |  |
| detected sequence I  （+599—+659） | *YGYGGGTATTTTTAATYGYGAGGGGTTGYGTTATTATYGGYGGTTGTTGGAGYGGTTGYGGGAGTTGGGYG* | 77-86 |
| mKL-CGI-Fseq-10 | GTGGTTATTTTGTATTATTGGGATTTGTTAT |  |
| detected sequence J  （+709—+777） | *AGYGTTTGTAGGATATTTATGGYGGATGGGTTAATYGYGTTTTGGTYGATTATTTTAGGGATTATGTYG* | 88-93 |
| mKL-CGI-Fseq-11 | GGTGGTTAGGTTAAGTATTGGATTATTA |  |
| detected sequence K  （+826—+885） | *TTGATAATTTTTAYGTGGTGGTTTGGTAYGGGTATGTT*  *ATYGGGYGTTTGGTTTYGGGYG* | 96-101 |

Note: CpG detection sites (TSS is "+1"; first CpG site# is "-42")

TableS8: Nested PCR for HK-2cells

1. PCR primers

| Name | Sequence | Amplified fragments（bp） |  |
| --- | --- | --- | --- |
| hKL-CGI-F1.1 | | ATTAAAAGGTAATAGTAAAAGGGAGAGT |  |
| hKL-CGI-F2.1 | | AGGTAATAGTAAAAGGGAGAGTAAAATT |  |
| hKL-CGI-R1.1 | | AACCTTCAACACCCCAACCATATAACTCCRACTAAACATTACTAAAACCA | -717—-491 |
| hKL-CGI-F1.2 | | TGGTTTTAGTAATGTTTAGTYGGAGT |  |
| hKL-CGI-F2.2 | | GGAGAAAAGTGAGAGTAGGTGTTT |  |
| hKL-CGI-R1.2 | | AACCTTCAACACCCCAACCATATAAACACCTATTTCTCCCAACTCC | -471—-256 |
| hKL-CGI-F1.3 | | GGTAGGTAAAGAGAATGAATTTGAG |  |
| hKL-CGI-F2.3 | | GGAGTTGGGAGAAATAGGTGTT |  |
| hKL-CGI-R1.3 | | AACCTTCAACACCCCAACCATATAAACTCCRCTAACAATAATTACCTAC | -277—-110 |
| hKL-CGI-F1.4=F2.4 | | GTAGGTAATTATTGTTAGYGGAGTT |  |
| hKL-CGI-R1.4 | | AACCTTCAACACCCCAACCATATACAACAACACCAACAACAACRACAAC | -131—+72 |
| hKL-CGI-F1.5 | | GTTGTYGTTGTTGTTGGTGTTGTTG |  |
| hKL-CGI-F2.5=F1.5 | | GTTGTYGTTGTTGTTGGTGTTGTTG |  |
| hKL-CGI-R1.5 | | AACCTTCAACACCCCAACCATATATACTACCAACCRCCCTCRATCT | +48—+245 |
| hKL-CGI-F1.6 | | AGATYGAGGGYGGTTGGTAGTA |  |
| hKL-CGI-R1.6 | | AACCTTCAACACCCCAACCATATAAACRTTATTATAACTATCRCTAACTAC | +224—+370 |
| hKL-CGI-F1.7 | | GTAGTTAGYGATAGTTATAATAAYGTT |  |
| hKL-CGI-R1.7 | | AACCTTCAACACCCCAACCATATACTAAAACAAATCCCAATAATACAAAATAAC | +370—+594 |
| hKL-CGI-F1.8 | | GTTATTTTGTATTATTGGGATTTGTTTTAG |  |
| hKL-CGI-R1.8 | | AACCTTCAACACCCCAACCATATACTAACCCCTCRCACTCACCAA | +565—+837 |
| hKL-CGI-R2-Biotin | | TATATGGTTGGGGTGTTGAAGGTT |  |

(2) PCR：

First round PCR

| System | Components | Volume |
| --- | --- | --- |
| 10μl | 2*Taq（No.PT102-01, 公司） | 5μl |
|  | F（10μM） | 0.1μl |
|  | R（10μM） | 0.1μl |
|  | Temp | 1.5μl |
|  | ddH_2_O | 3.3μl |
| PCR parameters | 98°1min30s；98°15s，58°30s，72°30s🡪（35cycles）；72°5min；4°+∞ | |

Second round PCR

| System | Components | Volume |
| --- | --- | --- |
| 50μl | 2*Taq | 15μl |
|  | F (10μM) | 0.5μl |
|  | hKL-CGI-R2-Biotin（10μM） | 0.5μl |
|  | Temp（the products of first round PCR） | 7μl |
|  | ddH_2_O | 27μl |
| PCR parameters | 98°1min30s；98°15s，58°30s，72°30s🡪（38cycles）；72°5min；4°+∞ | |

TableS9: Pyrosequencing primers and detection sequence of HK-2 cells

| Name | Sequence | CpGsite# |
| --- | --- | --- |
| hKL-CGI-Fseq1.1=F2.1 | AGGTAATAGTAAAAGGGAGAGTAAAATT |  |
| detected sequence A  (-689— -615) | *TTAGTTTTAAYGTAATTTATAAATTTATTTTGTTTTTTTYGAAAGAGGGGYGYGGGTGGGYGYGTTTTTTYGYGA* | 1-7 |
| hKL-CGI-Fseq1.2 | GAGTATTTTATTTAAGGGGGAATTT |  |
| detected sequence B  (-591— -494) | *TTTTTAGYGTAYGGYGAAGTTTTTTTTYGGTTGTTTTATTTGGTAGTTTTTTTAGGATTTYGGTTAGTTTTTAATTGGTTTTAGTAATGTTTAGTYGG* | 8-13 |
| hKL-CGI-Fseq1.2=F2.2 | GGAGAAAAGTGAGAGTAGGTGTTT |  |
| detected sequence C  (-447— -359) | *TTTTAGYGGYGYGTTTYGTTAGGGTTYGGTAGGATTTYGTTTTTAAGTYGGGGAAAGTTGGTYGGYGTTTTTTTTTTTYGAYGAAGTYG* | 15-26 |
| hKL-CGI-Fseq2.2 | GTTTTAGGGTTGTTTTTAGAGGA |  |
| detected sequence D  (-336— -278) | *YGYGYGGTAGGTAAAGAGAATGAATTTGAGYGTTTAYGAAAYGTTTTGTAYGGTTTTYG* | 27-34 |
| hKL-CGI-Fseq1.3=F2.3 | GGAGTTGGGAGAAATAGGTGTT |  |
| detected sequence E  (-255— -165) | *TTTTTTYGAYGTTYGYGGGYGAYGTTTGTYGTATTTTGTTYGTTGTYGYGTTTTTTTYGGGTATTTTTYGTTTTYGGYGTTTTTGTTTTYG* | 35-49 |
| hKL-CGI-Fseq-1.4=F1.4 | GTAGGTAATTATTGTTAGYGGAGTT |  |
| detected sequence F  (-106— -28) | *YGTYGGGGAGYGGGGGTGGGYGYGTYGGYGGTGGGYGGGYGGGYGYGGYGGGGYGYGGGTATAAAGGGGYGYGGYGYG* | 54-71 |
| hKL-CGI-Fseq2.4 | GGGGTTTYGGAGTTTGGTTT |  |
| detected sequence G  (-9— +54) | *TYGYGTAGTATGTTYGTTAGYGTTTYGTYGYGTYGTTYGYGGTYGTYGTYGTYGTYGTTGTYG* | 73-88 |
| hKL-CGI-Fseq1.5=F1.5 | GTTGTYGTTGTTGTTGGTGTTGTTG |  |
| detected sequence H  (+73— +144) | *GGTTTGGGYGGTYGTYGTTTGYGTGYGGAGTYGGGYGAYGGYGYGTAGATTTGGGTTYGTTTTTYGYGGTTT* | 89-101 |
| hKL-CGI-Fseq2.5 | GYGTAGATTTGGGTTYGTTT |  |
| detected sequence I (+135—+217) | *TTYGYGGTTTTTTGTTTTYGAGGTYGYGGGTTTTTTTTAGGGTATTTTTTTYGAYGGTTTTTTTTGGGTYGTGGGTAGYGTYG* | 100-109 |
| hKL-CGI-Fseq1.6=F1.6 | AGATYGAGGGYGGTTGGTAGTA |  |
| detected sequence G (+246—+319) | *GTAYGGTAAGGGTGYGTTTATTTGGGATAYGTTTATTTATTATTTTTTGGTATTTTYGGGAGATTTTYGGAAYG* | 112-117 |
| hKL-CGI-Fseq2.6 | TTGGTATTTTYGGGAGATTTT |  |
| detected sequence K (+313—+394) | *YGGAAYGTTAGTTTGTYGTTGGGYGTTTYGTYGTYGTTGTAGTTYGTTATYGGGGAYGTAGTTAGYGATAGTTATAATAAYG* | 116-127 |
| hKL-CGI-Fseq1.7=F1.7 | GTAGTTAGYGATAGTTATAATAAYGTT |  |
| detected sequence L (+397—+464) | *TTTYGYGATAYGGAGGYGTTGYGYGAGTTYGGGGTTATTTATTATYGTTTTTTTATTTYGTGGGYGYG* | 127-138 |
| hKL-CGI-Fseq-2.7 | TGGGYGYGAGTGTTTTTTAATGG |  |
| detected sequence M (+480—+562) | *TAGYGYGGGYGTTTTTAATYGYGAGGGGTTGYGTTATTATYGGYGTTTGTTGGAGYGGTTGYGGGAGTTGGGYGTGTAGTTYG* | 139-150 |
| hKL-CGI-Fseq1.8=F1.8 | GTTATTTTGTATTATTGGGATTTGTTTTAG |  |
| detected sequence N (+595—+685) | *YGTTTGTAGGAYGTTTAYGGYGGTTGGGTTAATYGYGTTTTGGTYGATTATTTTAGGGATTAYGYGGAGTTTTGTTTTYGTTATTTYGGYG* | 151-162 |
| hKL-CGI-Fseq2.8 | GGTTAGGTTAAGTATTGGATTATTAT |  |
| detected sequence O (+711—+804) | *YGATAATTTTTAYGTGGTGGTTTGGTAYGGTTAYGTTATYGGGYGTTTGGTTTTYGGTATTYGGGGTAGTTYGYGGTTYGGGTATTTGGTGGY* | 163-174 |

Note: CpG detection sites (TSS is "+1"; first CpG site# is "-650")

**Table S10** Changes in Body Weight, 24-hour urinary protein, urinary 8-iso-PGF2a and 8-OHdG in mice after different treatment

|  | C57 group | db/db group | db/db+EGCG group |
| --- | --- | --- | --- |
| Body Weight (g) |  |  |  |
| Baseline | 24.9±0.9 | 33.3±1.8**^##^** | 33.1±2.1**^##^** |
| Week 8 | 36.1±2.5 | 45.5±2.1**^##^** | 44.9±3.7**^##^** |
| 24-hour urinary protein (mg) |  |  |  |
| Baseline | 1.2±0.5 | 5.8±1.0^##^ | 5.9±0.9^##^ |
| Week 8 | 1.1±0.6 | 10.5±1.4^##^ | 7.7±0.8^##^****** |
| Urinary 8-iso-PGF2α(ng/d)  Baseline  Week 8 | 37.9±8.9  38.8±6.6 | 85.2±8.3^##^  175.4±22.9^##^ | 86.7±11.2^##^  138.6±19.5^##^****** |
| Urinary 8-OHdG (ng/d) |  |  |  |
| Baseline | 13.1±4.6 | 38.5±8.4^##^ | 37.4±6.5^##^ |
| Week 8 | 12.8±5.3 | 82.7±6.9^##^ | 55.3±7.2^##^****** |

Note: C57 group, C57BLKS/J normal mice non-treated; db/db group, C57BLKS/J db/db mice non-treated; db/db+EGCG group, db/db mice treated with EGCG of 50 mg/kg/d; 8-iso-PGF2α, urinary 8-iso-prostaglandin F2α; 8-OHdG, 8-hyOdroxy-2’-deoxyguanosine; **^#^***P*<0.05, **^##^***P*<0.01 *vs* C57 group;******P*<0.05, *******P*<0.01 *vs* db/db group；Values are means ± SEM. n=16 in each group.

**Table S11** Changes in parameters of oxidative stress, and proinflammatory cytokines in kidney homogenates in mice after different treatment

|  | C57 group | db/db group | db/db+EGCG group |
| --- | --- | --- | --- |
| **Oxidative stress** |  |  |  |
| ROS (U/ml)  Baseline | 203.4±17.5 | 275.9±21.6^##^ | 276.6±19.8^##^ |
| Week 8 | 205.8±20.7 | 480.5±17.9^##^ | 353.7±20.5^##^****** |
| MDA (nM/mg protein)  Baseline  Week 8  CAT (U/mg protein)  Baseline  Week 8 | 14.5±2.2  14.9±3.8  26.8±3.1  24.1±2.6 | 17.1±2.1^#^  29.2±3.6^##^  21.7±2.4^#^  13.4±2.5^#^ | 16.8±1.5^#^  21.2±4.3^##^*****  21.1±2.6^#^  18.9±3.4^#^***** |
| SOD (U/mg protein) |  |  |  |
| Baseline | 61.3±5.7 | 49.4±6.1^#^ | 49.9±7.8^#^ |
| Week 8 | 60.8±6.5 | 30.6±7.4^##^ | 40.8±6.9^#^***** |
| 8-OHdG(pg/mg protein) |  |  |  |
| Baseline | 815.1±31.4 | 960.5±41.8^##^ | 973.3±45.6^##^ |
| Week 8 | 820.8±34.9 | 1675.3±45.2^##^ | 1491.4±48.3^##^****** |
| **inflammatory cytokine** |  |  |  |
| IL-1β (pg/ml) |  |  |  |
| Baseline | 15.3±2.6 | 28.4±5.5^#^ | 30.5±4.8^#^ |
| Week 8 | 14.8±3.2 | 46.7±3.4^##^ | 35.4±3.9^##^***** |
| IL-6 (pg/ml) |  |  |  |
| Baseline | 37.6±6.8 | 55.2±7.1^#^ | 53.3±6.4^#^ |
| Week 8 | 42.5±8.9 | 70.7±8.5^#^ | 62.2±7.3^#^***** |
| TNF-α (pg/ml) |  |  |  |
| Baseline | 19.7±2.3 | 53.4±4.6^#^ | 35.2±4.1^#^ |
| Week 8 | 18.8±3.5 | 57.6±5.8^##^ | 33.4±3.7^##^***** |

Note: C57 group, C57BLKS/J normal mice non-treated; db/db group, C57BLKS/J db/db mice non-treated; db/db+EGCG group, db/db mice treated with EGCG of 50 mg/kg/d; ROS, reactive oxygen species; SOD, Superoxide Dismutase; CAT, catalase; MDA, malondialdehyde; 8-OHdG, 8-hyOdroxy-2’-deoxyguanosine; IL-1β, interleukin-1β; IL-6, interleukin-6; TNF-α, tumor necrosis factor-α. **^#^***P*<0.05, **^##^***P*<0.01 *vs* C57 group;******P*<0.05, *******P*<0.01 *vs* db/db group；Values are means ± SEM. n=16 in each group.

**Table S12** Changes in parameters of oxidative stress, and proinflammatory cytokines in HK-2 cell after different treatment

|  | NG | HG | HG+EGCG | HG+klotho KO | HG+klotho KO+EGCG | HG+klotho OE |
| --- | --- | --- | --- | --- | --- | --- |
| Oxidative stress | |  |  |  |  |  |
| ROS(U/ml) | 62.4±5.9 | 92.3±10.2 | 78.3±8.6****** | 121.7±13.9****** | 106.5±18.5****** | 71.1±12.4****** |
| SOD  (U/mg protein) | 28.4±2.3 | 15.1±3.9 | 24.3±2.8****** | 11.7±3.6***** | 10.9±4.1***** | 26.5±3.4****** |
| MDA (nM/mg protein) | 1.48±0.57 | 6.17±1.51 | 4.25±1.49***** | 7.85±1.67***** | 7.69±0.93***** | 3.95±0.82***** |
| inflammatory cytokine | |  |  |  |  |  |
| TNF-α (pg/ml) | 210.7±42.8 | 617.8±76.5 | 328.4±39.3****** | 719.6±98.5****** | 694.9±81.4***** | 356.2±63.1****** |
| IL-6 (ng/ml) | 1.13±0.42 | 6.24±0.93 | 3.57±0.75****** | 8.15±1.67***** | 7.76±1.21***** | 3.15±0.72****** |
| IL-1β (pg/ml) | 100.2±31.8 | 326.9±54.6 | 213.5±36.5****** | 459.3±52.7****** | 431.4±37.3****** | 137.8±45.4****** |

NG：normal glucose; HG: high glucose; klotho KO: klotho gene knockout; klotho OE: klotho overexpression; ******P*<0.05, *******P*<0.01 *vs* HG group；Values are means ± SEM


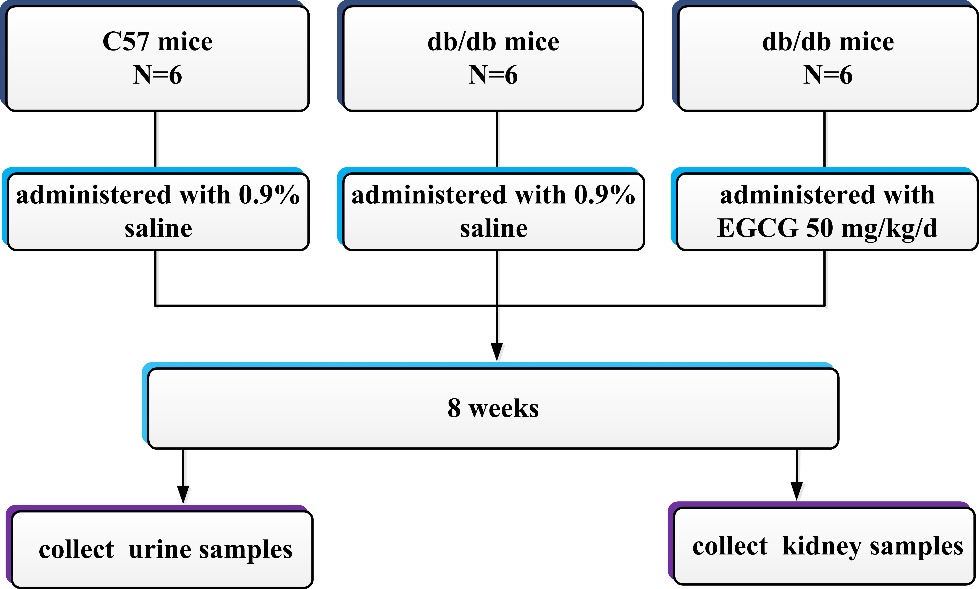


FigureS8: A graphical illustration of the mouse treatment plan

Normal group (non-treated C57 mice): C57 mice were intragastrically administered with 0.9% saline for 8 weeks; Control group (non-treated db/db mice): db/db mice were intragastrically administered with 0.9% saline for 8 weeks; EGCG group (db/db mice + EGCG): db/db mice were intragastrically administered with EGCG 50 mg/kg/d for 8 weeks;
